# Supplementary material for: Marker Identification of the Grade of Dysplasia of Intraductal Papillary Mucinous Neoplasm in Pancreatic Cyst Fluid by Quantitative Proteomic Profiling
Source: Cancers (Basel). 2020 Aug 23;12(9):2383. doi: 10.3390/cancers12092383 (PMC7565268; doi:10.3390/cancers12092383)
Supplement: Supplementary file 1 [file cancers-12-02383-s001.zip › cancers-911666-suppl - final/cancers-911666-suppl-proof.docx]

Supplementary Materials

Marker Identification of the Grade of Dysplasia of Intraductal Papillary Mucinous Neoplasm in Pancreatic Cyst Fluid by Quantitative Proteomic Profiling

Misol Do, Hongbeom Kim, Dongyoon Shin, Joonho Park, Haeryoung Kim, Youngmin Han, Jin-Young Jang and Youngsoo Kim

1. Supplementary Materials and Methods

1.1. Cell culture

Human pancreatic cancer cell lines (PANC-1, Mia PaCa-2, and BxPC3) were purchased from American Tissue Cell Culture (ATCC, Manassas, VA, USA). PANC-1 and Mia PaCa-2 cells were cultured in 100-mm dishes with Dulbecco’s Modified Eagle’s Medium (DMEM), supplemented with 10% heat-inactivated fetal bovine serum (FBS), 100 U/mL penicillin, and 100 mg/mL streptomycin, at 37°C in a humidified 5% CO2 atmosphere. BxPC3 cells were cultured with Roswell Park Memorial Institute (RPMI) medium with the same supplements under identical conditions.

1.2. Enrichment of secreted proteins from conditioned media

Six cell culture dishes, each of which contained 2 × 106 cells, were washed 3 times with phosphate-buffered saline (PBS) and incubated with serum- and phenol red-free media for 24 h. The conditioned media was collected and centrifuged (3000 rpm, 3 min, 4°C) to remove floating cells and cellular debris. The supernatant was concentrated with 3K Amicon Ultra centrifugal filters (Millipore, Billerica, MA, USA) after being passed through a 0.22-µm-pore membrane. The protein concentration of the concentrated conditioned media was measured using a bicinchoninic acid (BCA) reducing agent compatibility assay kit (Thermo Fisher Scientific, Waltham, MA, USA). All samples were stored at -80°C.

1.3. Western blot for confirming the enrichment of secreted proteins

Protein samples, containing 30 µg of secreted proteins from conditioned media and cell lysate, were mixed with 5X SDS loading dye (250 mM Tris-Cl, pH 6.8, 10% SDS, 50% glycerol, 0.5 M DTT, 0.1% bromophenol blue). An equal volume of each sample was loaded onto 12% SDS-PAGE gels and transferred to polyvinylidene fluoride (PVDF) membranes (Hybond-P, GE Healthcare, Pittsburgh, PA). All membranes were blocked with 5% BSA for 2 h at RT and incubated overnight at 4°C with primary antibodies: mouse monoclonal anti-α tubulin (sc-5286, Santa Cruz Biotech., CA, USA) and mouse monoclonal anti-β actin (sc-517582, Santa Cruz Biotech., CA, USA) at 1:1000. The membranes were washed 5 times with a mixture of Tris-buffered saline and Tween-20 (TBS-T) for 10 minutes each. The membranes were then incubated with HRP-conjugated secondary antibodies: anti-mouse (ab6789, Abcam, Cambridge, UK) at 1:5000 for 2 h at RT. The membranes were incubated with ECL solution (West-Q chemiluminescent substrate Kit-plus, GenDEPOT, TX, USA), and bound antibodies were visualized on a LAS-4000 (Fujifilm, Tokyo, Japan).

1.4. Protein isolation from cell pellets and secreted protein

PANC1, Mia Paca-2, and BxPC3 cells pellets were rinsed with cold PBS and homogenized by sonication for 30 s (Sonics & Materials Inc., Newtown, CT, USA) in lysis buffer (4% SDS, 1 mM TECP, 0.1 M Tris-Cl, pH 7.4). The samples were boiled in a water bath for 30 min at 100°C and then centrifuged (15,000 rpm, 20°C, 20 min) to remove cell debris. Next, 100 µg of proteins from the 3 cell types were pooled for further processing. The pooled cell lysate and 300 µg of each secreted protein were precipitated with cold acetone (Sigma-Aldrich, St. Louis, MO, USA) at a ratio of 1:5 (sample:acetone, v/v). The mixture was incubated overnight at -20°C after being vortexed thoroughly. The precipitate was centrifuged for 10 minutes at 15,000 rpm at 4°C, and the acetone was gently removed. After an additional rinse step with 500 µL cold acetone and centrifugation, the protein pellet was air-dried for 2 h and stored for digestion.

1.5. Pancreatic cyst fluid sample preparation

In cases in which the cyst fluid was too viscous to be extracted with a pipette, the cyst fluid was sonicated briefly prior to mucus removal in a 1.5-mL Eppendorf tube [1]. The samples were centrifuged (15,000 rpm, 20 min, 4°C) to separate the supernatant from the cellular debris and other solid contents. Only the supernatant was used in this study. The protein concentration was measured by BCA assay, and 20 µg of proteins from each sample were pooled for further fractionation. One hundred fifty micrograms of proteins from each sample were precipitated with cold acetone in the same manner as the cell pellets.

1.6. Protein digestion and desalting

The acetone-precipitated samples (1 pooled cyst fluid, 30 individual cyst fluids, 1 pooled cell lysate, secreted proteins of 3 cell lines) were mixed with 30 µL SDT lysis buffer (4 % SDS, 0.1 M DTT, 0.1 M Tris-Cl, pH 7.4). After being vortexed gently, the mixture was boiled for 30 min at 100°C to denature the proteins. The denatured samples were mixed with 300 µL 0.22-µm-pore filtered UA buffer (8 M urea, 0.1 M Tris-Cl, pH 8.5) and then transferred to a 30-kDa centrifugal filter (Millipore, Billerica, MA, USA). The sample was then centrifuged 3 times (14,000 g, 15 min, 20°C) to remove SDS. The washed samples were incubated in 200 µL 50 mM iodoacetamide (IAA) in UA buffer at room temperature (RT) for 1 h to alkylate the reduced cysteine. After exchanging the UA buffer with 40 mM ammonium bicarbonate (ABC), the samples were digested with 0.1 µg/µL trypsin at a ratio of 1:50 (enzyme:substrate, wt/wt) for 18 h at 37°C.

The pooled samples were subjected to a second digestion step [1,2]. After being transferred to a new centrifuge tube, the filters were washed sequentially with 200 µL UA buffer once and 300 µL 40 mM ABC twice. Then, the proteins were digested with 0.1 µg/µL trypsin (trypsin:sample ratio of 1:100, wt/wt). The 30 individual cyst fluid digests were measured by tryptophan fluorescence assay to estimate the amounts of peptides. Equal amounts of peptides were acidified and desalted with homemade StageTips as described [3,4]. In contrast to the individual samples, all pooled samples were desalted without conserving any spare volume. The desalted samples were then lyophilized to dryness in a speed-vacuum centrifuge and stored at -80°C until fractionation and analysis.

1.7. High-pH reverse-phase peptide fractionation

A total of 5 library samples (pooled cyst fluid; pooled cell lysate; and the secreted proteins of PANC-1, Mia PaCa-2, and BxPC3 cells) were further fractionated on a modified Stage-tip column in a high-pH environment to generate a peptide library [2,5]. The desalted peptides from the first digest were reconstituted in 200 µL loading solution (15 mM ammonium hydroxide, pH 10, 2% acetonitrile) and separated on a pipette-based RP microcolumn, prepared by plugging the bottom of a 200-µL pipette tip with C18 Empore disk membrane (3M, St. Paul, MN, USA) and filling the tip with POROS 20 R2 resin. After 3 rinses each of 100% methanol, 100% acetonitrile (ACN), and loading buffer, the dissolved peptides were loaded onto the column and eluted into 20 fractions on a discontinuous ACN gradient (2, 5, 7.5, 10, 12.5, 15, 17.5, 20, 22.5, 25, 27.5, 30, 32.5, 35, 40, 50, 60, 70, 80, and 100%). These 20 fractions were concatenated into 6 fractions with varying hydrophobicities to optimize coverage and liquid chromatography-tandem mass spectrometry (LC-MS/MS) run time. The 6 fractions were dried in a vacuum centrifuge and stored at -80°C until LC-MS/MS analysis.

1.8. LC-MS/MS analysis

The peptides were analyzed by a Q Exactive mass spectrometer that was equipped with an EASY-Spray ion source (Thermo Fisher Scientific, Waltham, MA, USA), coupled to an Easy-nano LC 1000 (Thermo Fisher Scientific, Waltham, MA, USA), following our established protocol [1,6]. The peptide samples were separated on a 2-column setup that comprised a trap column (75 µm I.D. x 2 cm, C18 3.0 µm, 100 Å) and an analytical column (Easy-Spray Pepmap RSLC, 75 µm I.D. x 50 cm, C18 2.0 µm, 100 Å).

An ACN gradient (6% to 40%) run of 180 minutes was achieved by mixing solvent A (2% ACN and 0.1% v/v formic acid) and solvent B (100% acetonitrile and 0.1% v/v formic acid) in varying proportions. Peptides that were eluted from the analytical column were ionized at a spray voltage of 2.0 kV in positive ion mode. MS1 spectra were collected in data-dependent acquisition (DDA) mode using a top 15 method with a resolution of 70,000 at m/z 200 with a mass range of 350-1700 m/z. The 15 most abundant ions were fragmented by higher-energy collisional dissociation (HCD) with a normalized collision energy (NCE) of 27 at a resolution of 17,500 at m/z 200. The maximum ion injection times for the survey and MS/MS scans were 20 ms and 80 ms, respectively. The dynamic exclusion was set to 30 s to prevent repeated sequencing.

1.9. Raw data search

All raw MS files (120 files) from the Q Exactive were processed in MaxQuant, version 1.6.0.16 [7] with the built-in Andromeda search engine [8] against the Uniprot human database (88,717 entries, version from December 2014), containing the forward and reverse amino acid sequences. In accordance with the established target-decoy search procedures [9], the search results were filtered at a false discovery rate (FDR) < 1% for identifying peptides, modification sites, and proteins.

The database search was performed with the following parameters: digestion mode trypsin/P; main search and first search tolerances of 6 ppm and 20 ppm, respectively; tolerance of up to 2 missed cleavages; carbamidomethylation of cysteine as a fixed modification; oxidation of methionine and protein N-terminal acetylation as variable modifications; and peptide length of at least 6 residues. Peptides were assigned to protein groups by the principle of parsimony [10,11]. The principle was applied to reduce the number of ambiguous proteins, the identified peptides of which could belong to several proteins. The retention times of all raw files were aligned through the “match between runs” feature in MaxQuant, which allows the transfer of MS/MS spectra and sequence information within a retention time window of 0.7 min to other raw files that have insufficient MS/MS spectra to identify the sequences [12].

1.10. Label-free quantification and statistical analysis

Label-free quantification (LFQ) and statistical analysis were performed in MaxQuant (version 1.6.0.16) and Perseus (version 1.6.1.1), respectively, in accordance with our previous studies [1,2]. Normalized spectral protein intensity values (LFQ intensity values) were used to estimate the protein abundance [13]. LGD had 30 LFQ intensity values (10 biological replicates × 3 technical replicates), whereas the others had 15 LFQ intensities (5 biological replicates × 3 technical replicates). LFQ intensity values that were greater than 0 were deemed to be valid.

From the list of identified peptides, proteins that had 70% or more valid values in at least 1 histological group were used for the statistical analysis. This standard was used to exclude proteins that could not characterize at least 1 sample group. The missing values were estimated, based on a normal distribution (imputation width = 0.3, shift = 1.8) of log2-transformed LFQ intensities [1]. Student’s t-test (*p* < 0.05) was applied to identify significantly changed proteins. The seven comparative pairs that were used in the statistical analysis were LGD versus HGD (comparison 1), HGD versus invasive IPMN (comparison 2), and LGD versus invasive IPMN (comparison 3), SCN versus LGD (comparison 4), MCN versus LGD (comparison 5), SCN versus invasive IPMN (comparison 6), and MCN versus invasive IPMN (comparison 7). Proteins that were differentially expressed in at least 2 comparative pairs within comparisons 1 to 3 were considered initial biomarker candidates.

Subsequently, DEPs that increased or decreased consistently with greater malignancy of IPMN but not in the MCN and SCN groups were selected as final biomarker candidates of IPMN progression–i.e. the final candidates of Invasive IPMN must have been statistically significant in comparisons 6 and 7 and increased sequentially with greater IPMN malignancy. Similarly, the final candidates of LGD must have been statistically significant in comparisons 4 and 5 and decreased sequentially with greater IPMN malignancy.

1.11. Repeatability of CD55 enzyme-linked immunosorbent assay (ELISA)

The intraplate repeatability of the CD55 ELISA was calculated by measuring 3 replicates of 10 positive control (cyst fluid from 2 HGD and 8 invasive IPMN) and 11 negative control samples (cyst fluid from 4 LGD and 7 SCN) on a single plate. Three independent ELISA analyses were conducted at different times to analyze the interplate repeatability using a total of 21 samples (10 positive and 11 negative controls). The optical density (OD) was measured at a wavelength of 450 nm to calculate the standard deviation (SD) and coefficient of variation (CV) for each sample.

1.12. Western blot of CD55

Among the 70 cyst fluid samples that were used for ELISA, a portion of the samples (8 LGD, 4 HGD, 8 invasive IPMN, 5 MCN, and 5 SCN) were selected, based on their suitable protein concentration and remaining protein content, for further validation by western blot. Forty micrograms of cyst fluid samples, mixed with 5X SDS loading dye, were separated on 7% SDS-PAGE gels and transferred to PVDF membranes. The membranes were stained with Ponceau S (P7170, Sigma-Aldrich, MO, USA), blocked with 5% BSA for 2 h at RT, and incubated overnight at 4°C with CD55 rabbit monoclonal antibody (38730, Cell Signaling Tech., MA, USA) at 1:1000 [1]. The membranes were washed 5 times with TBS-T for 10 minutes each and then incubated with goat anti-rabbit IgG (HRP) (ab6721, Abcam, Cambridge, UK) at 1:1000 for 2 h at RT. All signals were detected by LAS-4000 (Fujifilm, Tokyo, Japan) after incubation with ECL solution (West-Q Chemiluminescent Substrate Kit-plus, GenDEPOT, TX, USA).

1.13. Immunohistochemistry

The immunohistochemical analysis was performed on 4-µm-thick unstained sections, cut from formalin-fixed paraffin-embedded tissues, with CD55 rabbit monoclonal antibody (31759, Cell Signaling Tech., MA, USA) at 1:600 and myeloperoxidase (MPO) rabbit polyclonal antibody (A0398, Dako, Glostrup, Denmark) at 1:5000 on a BenchMark XT (Ventana Medical System, Tucson, AZ, USA) as described [14]. All immunohistochemical stains were reviewed by an expert hepatopancreaticobiliary pathologist (haeryoung.kim@snu.ac.kr, H. K.).

2. Supplementary Results

2.1. Cyst fluid sample characteristics

The demographics and clinical information of the study sample are described in Table 1. The histological composition of the 30 pancreatic cyst fluid samples consisted of LGD (n=10), HGD (n=5), invasive IPMN (n=5), MCN (n=5), and SCN (n=5). Among the 5 PCLs, there was no significant difference in composition, with the exception of cyst size and serum CEA and CA 19-9 levels, as measured by chemiluminescent microparticle immunoassay. Of the PCLs, the invasive IPMN patient group had the highest average concentrations of CEA and CA19-9 at 5.48 ± 6.82 mg/L and 90.28 ± 129.71 mg/L, respectively. The average CEA concentrations were similar between all groups, except for invasive IPMN, and increased gradually with the progression of IPMN dysplasia. Serum CA19-9 levels were generally higher in the more severe forms of IPMN, as were CEA levels. The average CEA level was approximately 4 times higher in invasive IPMN than in LGD and HGD. In addition, the average concentration of CA19-9 in invasive IPMN was approximately 8 times that of LGD and 4 times that of HGD. A baseline of 3 cm was used to classify cyst sizes [15,16]. The MCN group had the largest average cyst size (7.50 ± 2.18 cm), followed by invasive IPMN (5.74 ± 3.69 cm).

2.2. Reproducibility of label-free quantification data

All CV values for the sums of log2-transformed LFQ intensities across technical replicates of individual samples were less than 20% (0.959% to 13.279%) (Table S3). These data support that the variance that is attributed to the sample injection and quantification was low. The median CVs of log2-transformed LFQ intensities between technical triplicates of each sample ranged from 0.555% to 3.564%, indicating that the label-free quantification of cyst fluid samples had high reproducibility (Figure S3). In addition, the average Pearson correlation coefficients of the technical triplicates ranged from 0.871 to 0.954 (Figure S4, Table S4). The high Pearson correlation coefficients between technical replicates of individual samples indicate that the reproducibility between replicates is high and that the data are suitable for statistical analysis.

2.3. Comparison with proteome databases and other researches

To examine the composition of pancreatic cyst fluid proteins, our data were compared with various proteome data from databases and past studies. The proteins that were identified in individual cyst fluid samples were compared with 3 public databases to screen for secreted proteins. As a result, 1578, 668, and 432 proteins were identified in SecretomeP, SignalP, and TMHMM, respectively (Figure S5A, Table S1) [17-19]. Secreted proteins accounted for 58.1% (1889 proteins) of the 3249 proteins that were identified in individual cyst samples (Figure S5B).

All protein accession numbers were mapped to gene symbols to compare them with 3 proteome databases: (1) the Human Plasma Protein Database, (2) the Human Protein Atlas (http://www.proteinatlas.org, June 11, 2018), and (3) the “core” proteome in Wilhelm et al [20]. A total of 3039 proteins were listed with their corresponding gene symbols after redundant genes were removed. When compared with the Human Plasma Proteome Database, 79.8% (2424) of the 3039 identified proteins were confirmed to be expressed in plasma or serum (Figure S5C, Table S1) [21,22]. Our dataset was compared with the Human Protein Atlas to estimate the percentage of pancreatic tissue-specific proteins. As a result, 2937 (96.6%) genes had evidence of corresponding mRNA entries, and 2665 (87.7%) genes had evidence of corresponding protein entries in the pancreas (Figure S5D, Table S1). Our data were compared with the core proteome in Wilhelm et al., which compiled 5 of the largest proteomic databases and extracted 11,578 human proteins that were ubiquitously expressed in all databases. The comparison found that 2714 (89.3%) of the identified proteins overlapped with those of the core proteome (Figure S5E) [20,23].

Our previous study identified 2992 proteins in pancreatic cyst fluid samples of IPMN patients by LC-MS/MS analysis. This study identified 5834 proteins, surpassing the earlier report in terms of proteome coverage (Figure S5F) [1]. In addition, the expression patterns of the marker candidates (AKR1B10, TFF1, SERPINA5, SERPINA4, MUC5AC, MUC2, TLN1, and TYMP) from our previous study were replicated here (Figure S6, Table S5).

2.4. Molecular characterization of DEPs of IPMN dysplasia

Gene ontology (GO), KEGG pathway analysis, and Ingenuity Pathway Analysis (IPA) were used to characterize the 364 DEPs obtained from the statistical analysis of IPMN dysplasia. A total of 364 proteins were subsequently subjected to gene ontology (GO) analysis and filtered using a *p* < 0.05. The top 8 biological process (BP) terms were significantly associated with molecular transport and malignancy, such as “vesicle-mediated transport,” “secretion,” “exocytosis,” “cell death,” and “cell motility.” In addition, cellular components (CCs) of the DEPs were primarily related to the extracellular compartment, indicating that the proteins in pancreatic cyst fluid are secreted mainly from the surrounding cells and originally reside in the extracellular matrix. The terms that were associated with peptidase activity were present at higher proportions in molecular function (MF), demonstrating that pancreatic cyst fluid contains many digestive enzymes generated from the pancreas (Figure S9, Table S8) [24]. According to the KEGG pathway enrichment (*p* < 0.05), all DEPs of IPMN dysplasia belonged to the “pancreatic secretion pathway” and “glycosylation/gluconeogenesis pathway” (Figure S9, Table S8).

IPA was conducted to better understand the association of DEPs with malignancy and their nature regarding pancreatic cyst fluid. A total of 216 and 247 DEPs from comparisons 1 (LGD vs HGD) and 3 (LGD vs invasive IPMN) were analyzed, respectively. To evaluate the biological functions that were associated with the DEPs of comparisons 1 and 3 and their activation levels, core analysis was conducted by using the protein accession numbers and their fold-change values (Figure S10A, B). As expected, the biological functions that were related to malignancy and molecular secretion had a higher rank among the biological functions that were associated with the DEPs of comparisons 1 and 3. The representative functions included “cellular movement,” “cancer,” “organismal injury and abnormalities,” “organismal development,” and “molecular transport,” which included such subcategories as “cell spreading,” “angiogenesis,” “secretion of molecules,” and “secretion of proteins.”

Heat maps of the comparative analysis were used to visualize the diseases and biological function terms across analyses simultaneously to detect trends and significant clusters (Figure S10C,D), allowing us to identify the diseases and biological functions that are predicted to increase or decrease similarly across comparisons 1 and 3. Consistent with our expectations, the biological functions that were related to malignancy (cell spreading, vasculogenesis, and cancer) and molecular secretion (secretion of molecules and secretion of proteins) were highly expressed in comparisons 1 and 3. In addition, the expression levels of malignancy-related terms and molecular secretion-related terms were higher in comparison 3 than in comparison 1, and “cell death of pancreatic cancer cell lines” decreased in comparison 3 (Figure S10C). Pancreas-specific diseases (chronic pancreatitis and poorly differentiated malignant pancreatic tumor) and cancer were significantly associated with the DEPs of comparison 3 (Figure S10D).

2.5. Repeatability of CD55 ELISA

The intraplate and interplate repeatability of the ELISA were calculated to evaluate the precision of the CD55 ELISA. The range of CV values of each control sample is summarized in Figure S12. The OD_450_ values of 3 replicates with 10 positive and 11 negative control samples were measured in a single ELISA plate to evaluate the intraplate repeatability. The OD_450_ values had CVs of between 0.363% and 12.663%, with an average CV value of 3.645% (Table S11). The OD_450_ value of each control sample was measured at different times to evaluate the interplate repeatability. The analysis yielded an average CV value of 7.130%, and individual CVs ranged from 1.378% to 19.846% (Table S11). The CV values for the intra-assay and inter-assay comparisons were less than 20%, indicating that the CD55 ELISA is highly reproducible and stable.

3. Supplementary Figures:


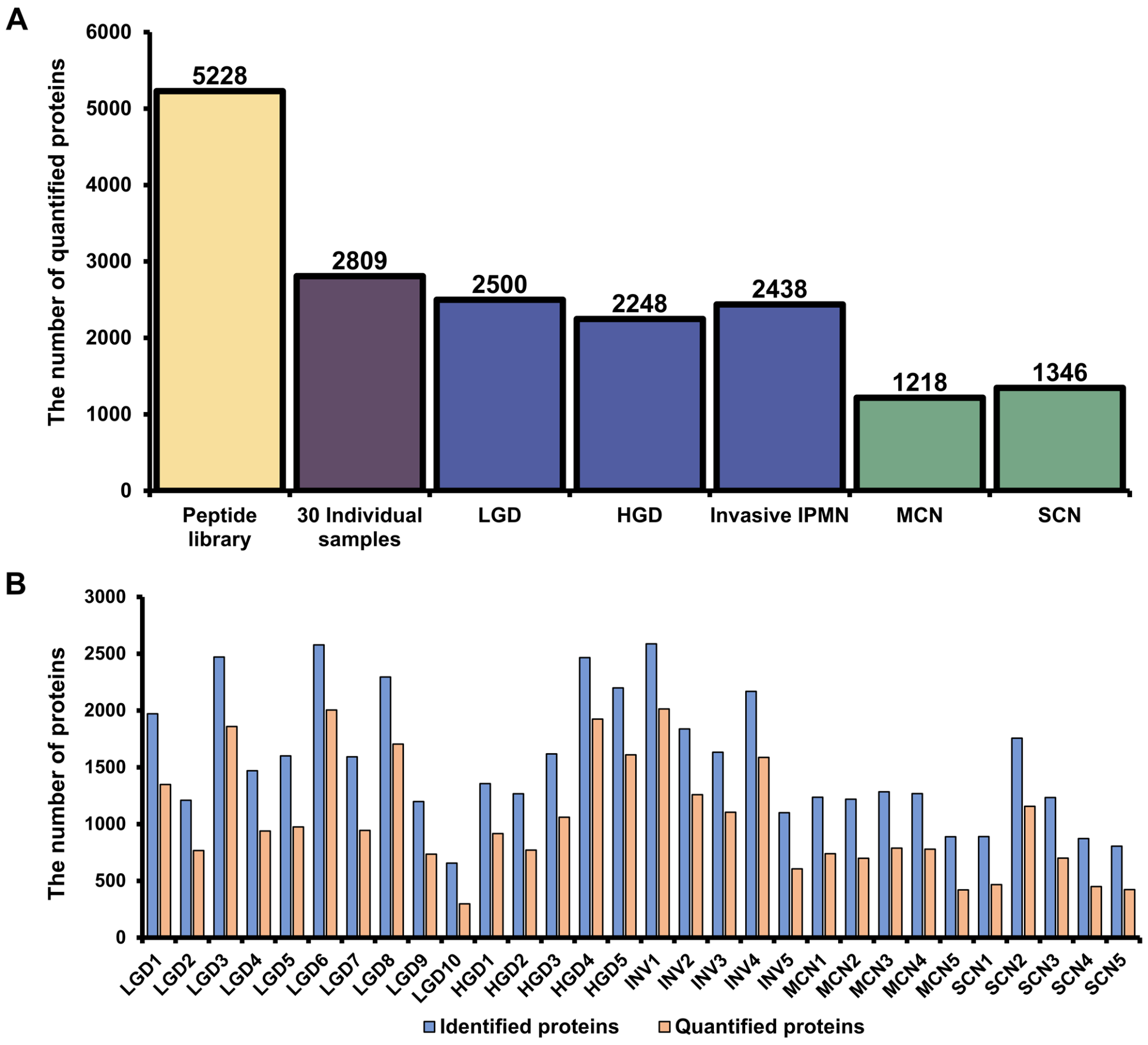


**Figure S1.** The number of identified and quantified proteins in individual samples and the peptide library. (**A**) The number of quantified proteins in the peptide library, the 30 individual samples, and each sample group (LGD, HGD, invasive IPMN, MCN, and SCN) is represented in a bar graph. (**B**) The number of identified and quantified proteins in each individual sample is indicated in a single bar graph. LGD, low-grade dysplasia; HGD, high-grade dysplasia; INV, invasive IPMN; MCN, mucinous cystic neoplasm; SCN, serous cystic neoplasm.


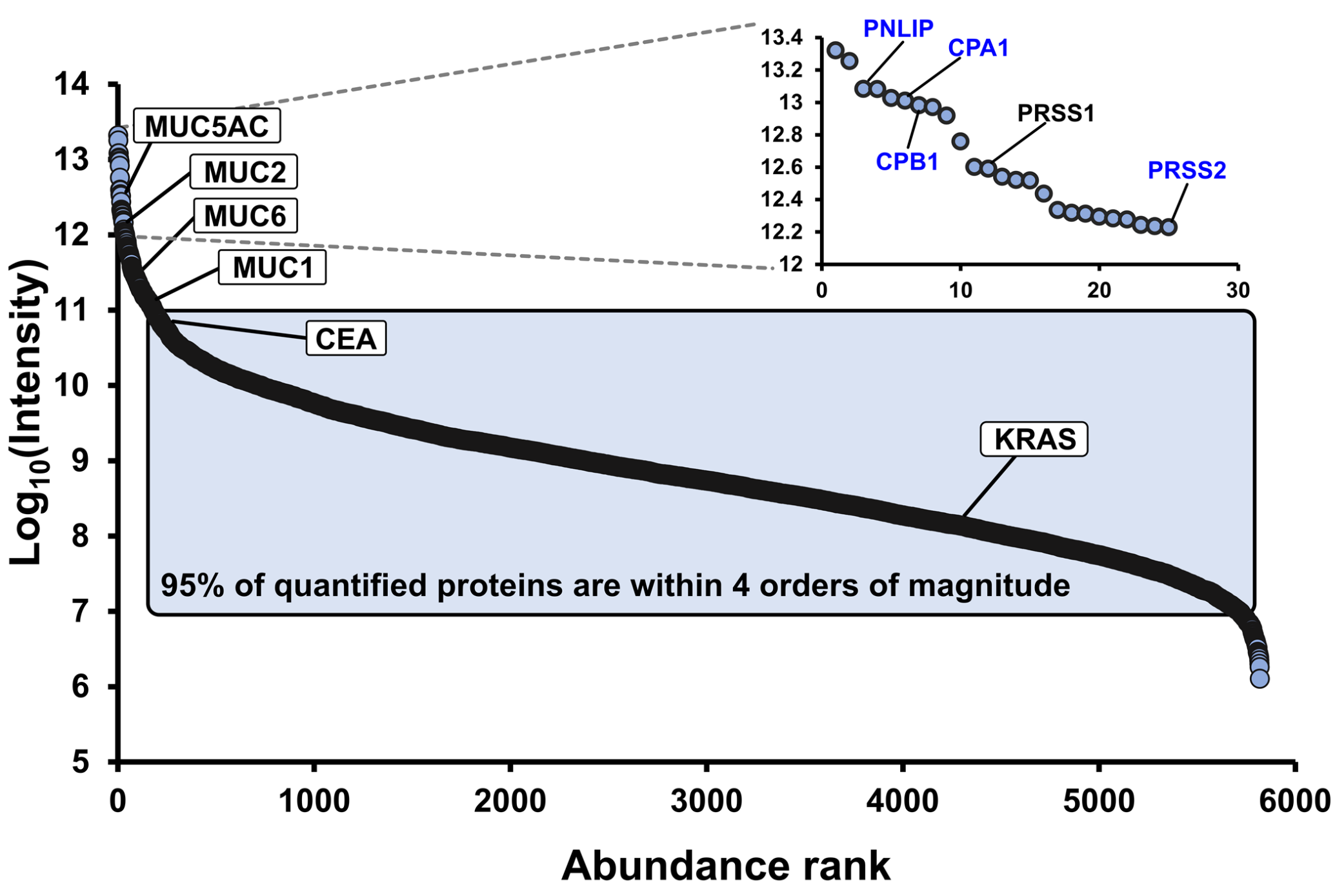


**Figure S2.** Dynamic range of quantified proteins. The expression levels of quantified proteins exceeded 7 orders of magnitude. Several markers of pancreatic cancer (MUC5AC, MUC2, and CEA) were high in abundance. Five pancreas-specific proteins (PNLIP, CPA1, CPB1, PRSS1, and PRSS2) were ranked in the top 25.


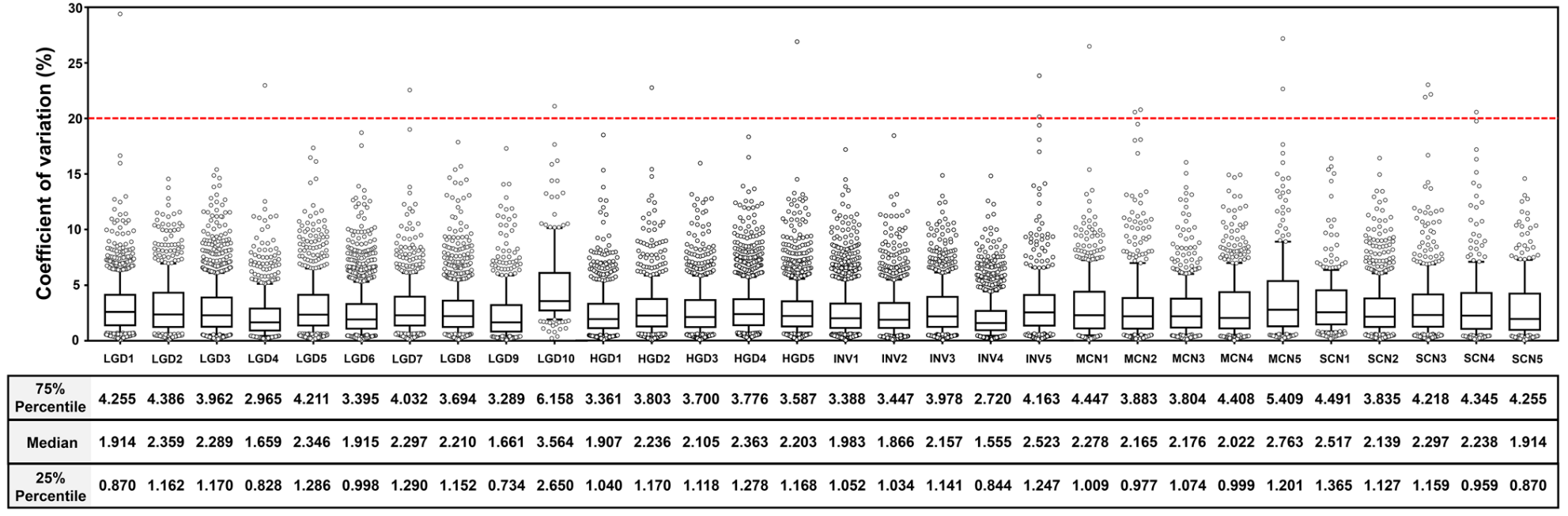


**Figure S3.** Coefficient of variation (CV%) values of technical triplicates in each individual sample. The median coefficient of variation (CV%) of log2-transformed LFQ intensity values between the technical triplicates in each individual is represented as box plots. All median CV values were less than 20%. LGD, low-grade dysplasia; HGD, high-grade dysplasia; INV, invasive IPMN; MCN, mucinous cystic neoplasm; SCN, serous cystic neoplasm.


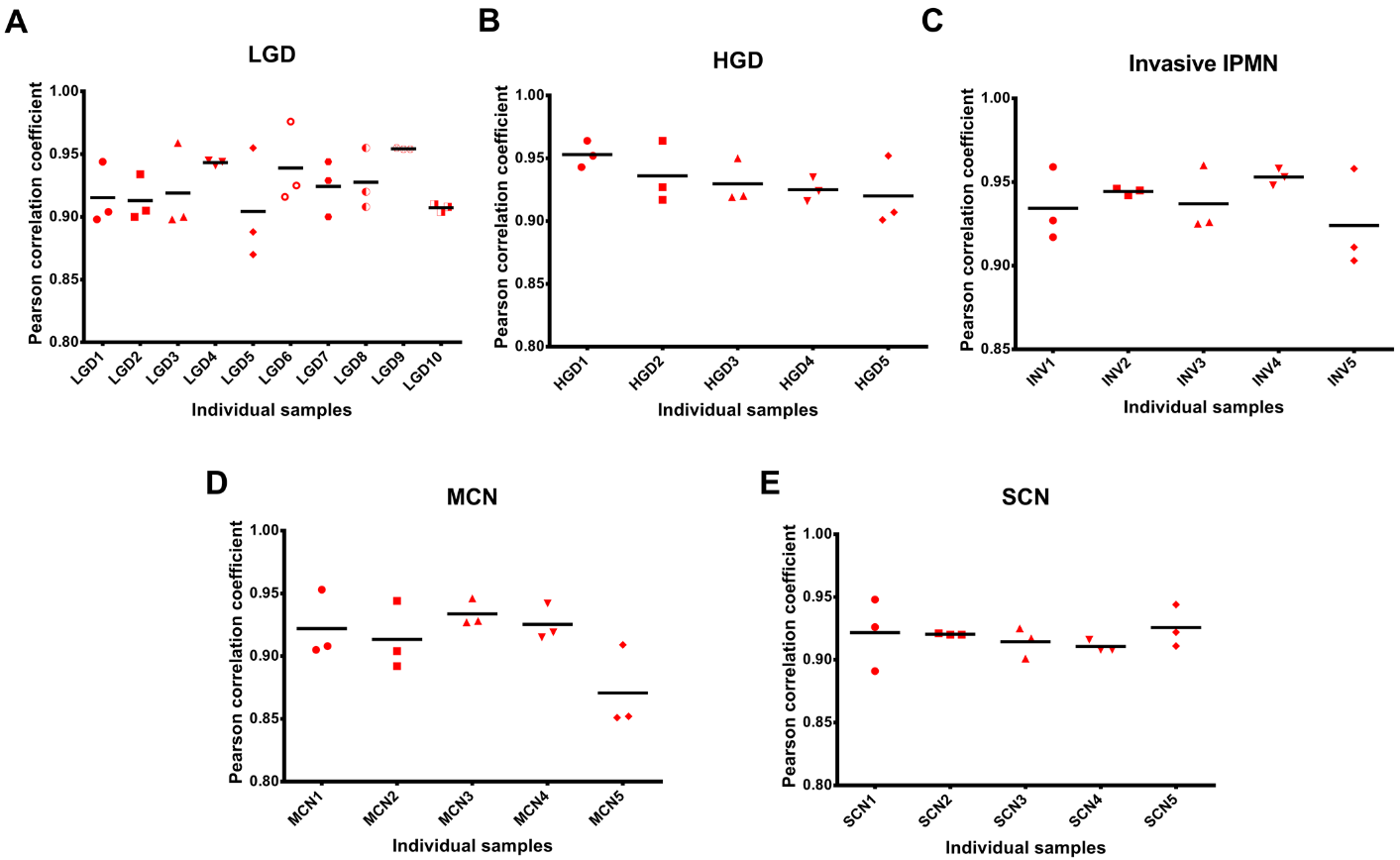


**Figure S4.** Pearson correlation coefficients between technical replicates in each sample group. Pearson correlation coefficients of technical replicates (TRs) in LGD (A), HGD (B), invasive IPMN (C), MCN (D), and SCN (E). The red markers represent Pearson correlation coefficient values of 3 comparisons (TR1 vs TR2, TR2 vs TR3, and TR1 vs TR3). The horizontal line represents the average of 3 Pearson correlation coefficient values in each individual sample. The average value of 3 Pearson correlation coefficients in each individual sample was larger than 0.870. LGD, low-grade dysplasia; HGD, high-grade dysplasia; INV, invasive IPMN; MCN, mucinous cystic neoplasm; SCN, serous cystic neoplasm.


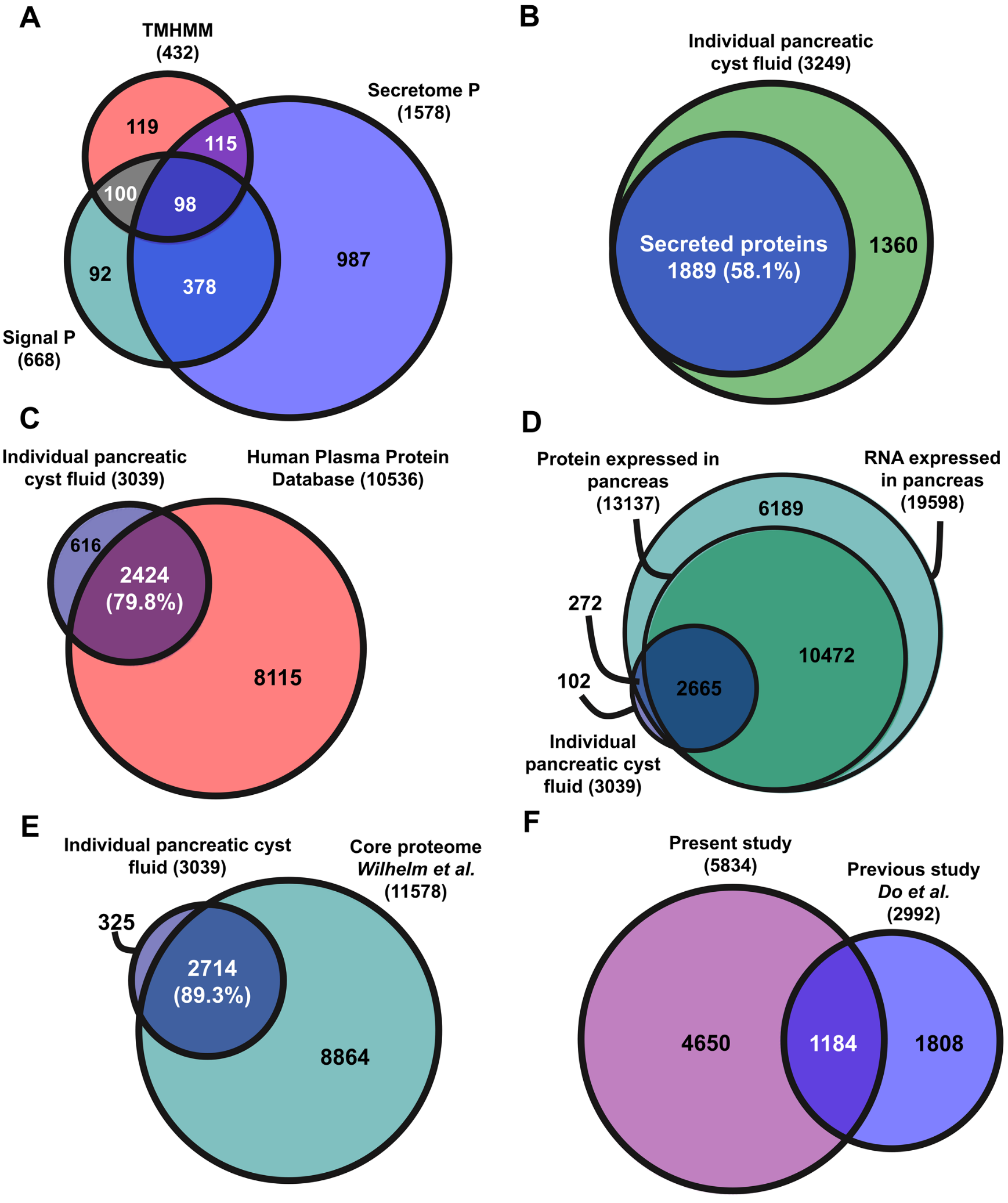


**Figure S5.** Comparative analysis with various proteome databases and other proteomic studies. (A) Of the 1889 secreted proteins, 1578, 668, and 432 were predicted to be secreted by SecretomeP, SignalP, and TMHMM, respectively. (B) In total, secreted proteins accounted for 58.1% (1889 proteins) of the 3249 proteins identified in cyst fluid. (C) In comparison with the Human Plasma Proteome Database, 2424 (79.8%) proteins were observed in plasma or serum. (D) Compared with the Human Protein Atlas, 2937 (96.6%) and 2665 (87.7%) proteins had evidence of corresponding mRNA and protein entries, respectively, in the pancreas. (E) In a comparative analysis with identified proteins in individual cyst samples and the core proteome in Wilhelm et al., 2714 (89.3%) proteins were found to be core proteins. (F) A total of 5834 proteins were identified in our dataset, which was approximately twice that of our previous study (Do et al.).


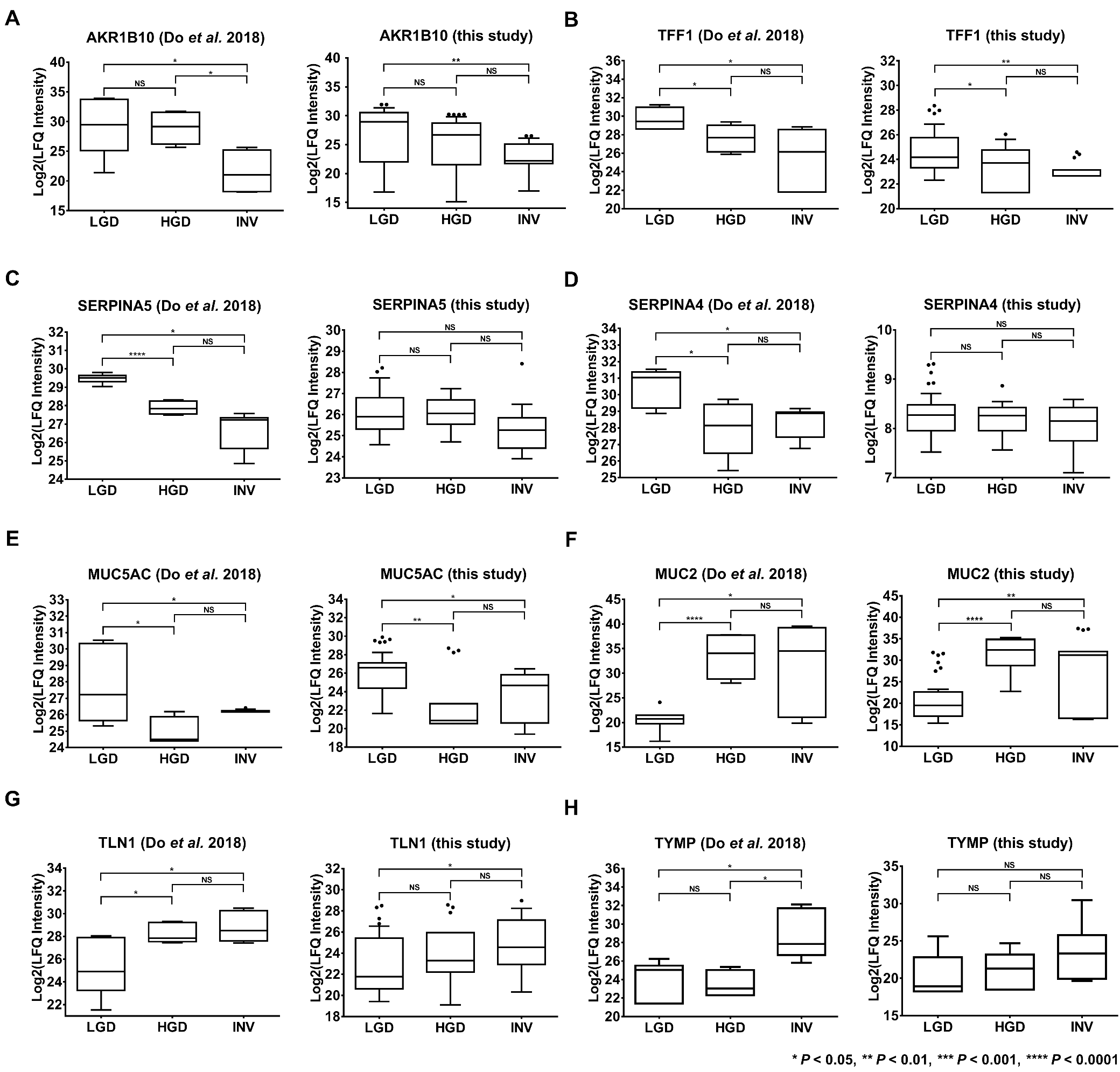


**Figure S6.** Comparison of the expression patterns of the final marker candidates between our previous and present studies. In comparison with our previous study, the protein expression patterns in IPMN dysplasia of this dataset were consistent with the 8 final marker candidates (AKR1B10, TFF1, SERPINA5, SERPINA4, MUC5AC, MUC2, TLN1, and TYMP) from our earlier study. LGD, low-grade dysplasia; HGD, high-grade dysplasia; INV, invasive IPMN; *, *p* < 0.05; **, *p* < 0.01; ***, *p* < 0.001; ****, *p* < 0.0001; NS, not available.


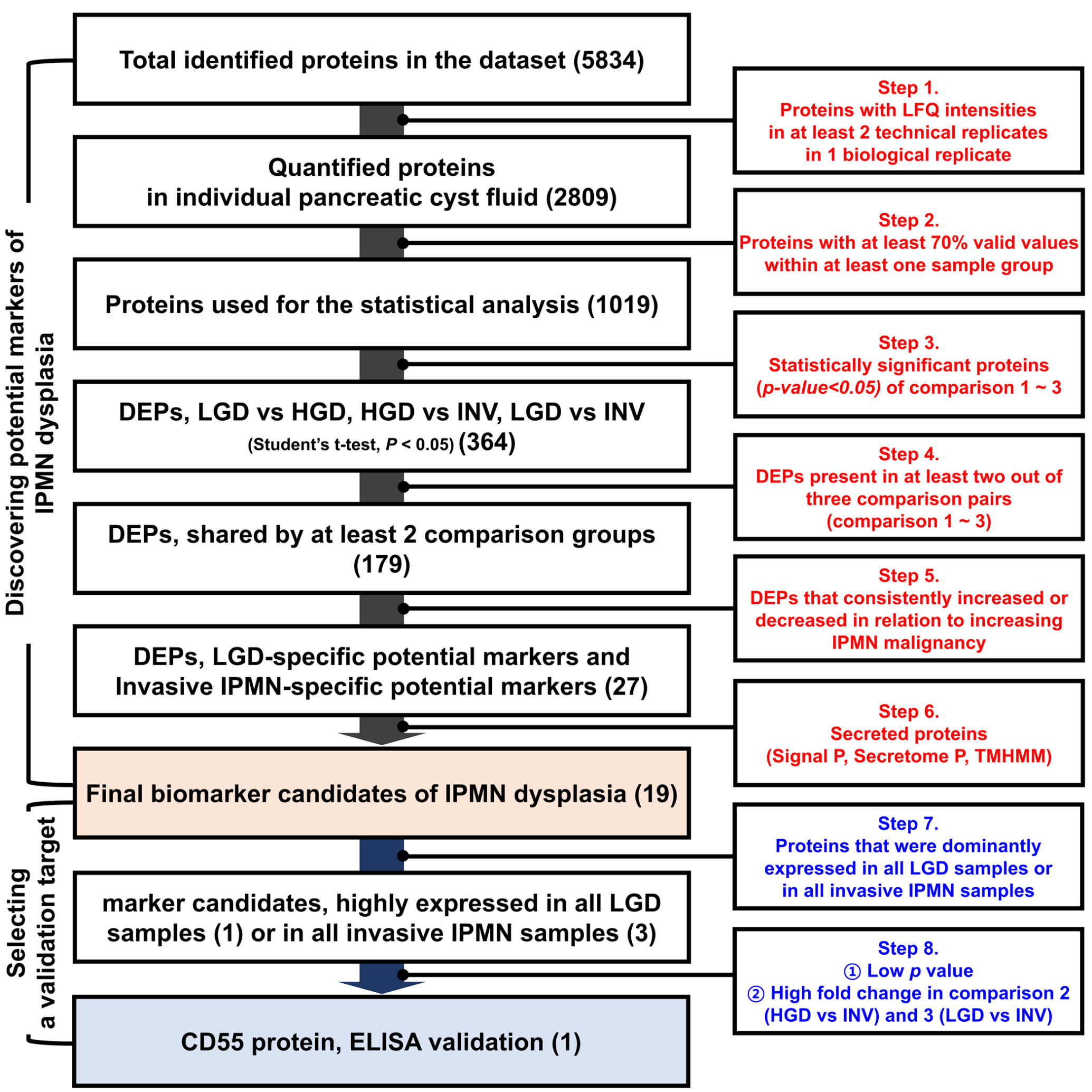


**Figure S7.** Flowchart of the discovery of potential markers of IPMN dysplasia. Potential markers of the histological grades of IPMN were discovered following this logically sound step-by-step procedure. The procedure is composed of 6 steps for discovering potential markers of IPMN dysplasia and 2 steps for selecting a target for validation. The 70% valid value criterion in the step 2 was applied to eliminate proteins that failed to represent any histological group. LGD, low-grade dysplasia; HGD, high-grade dysplasia; INV, invasive IPMN.


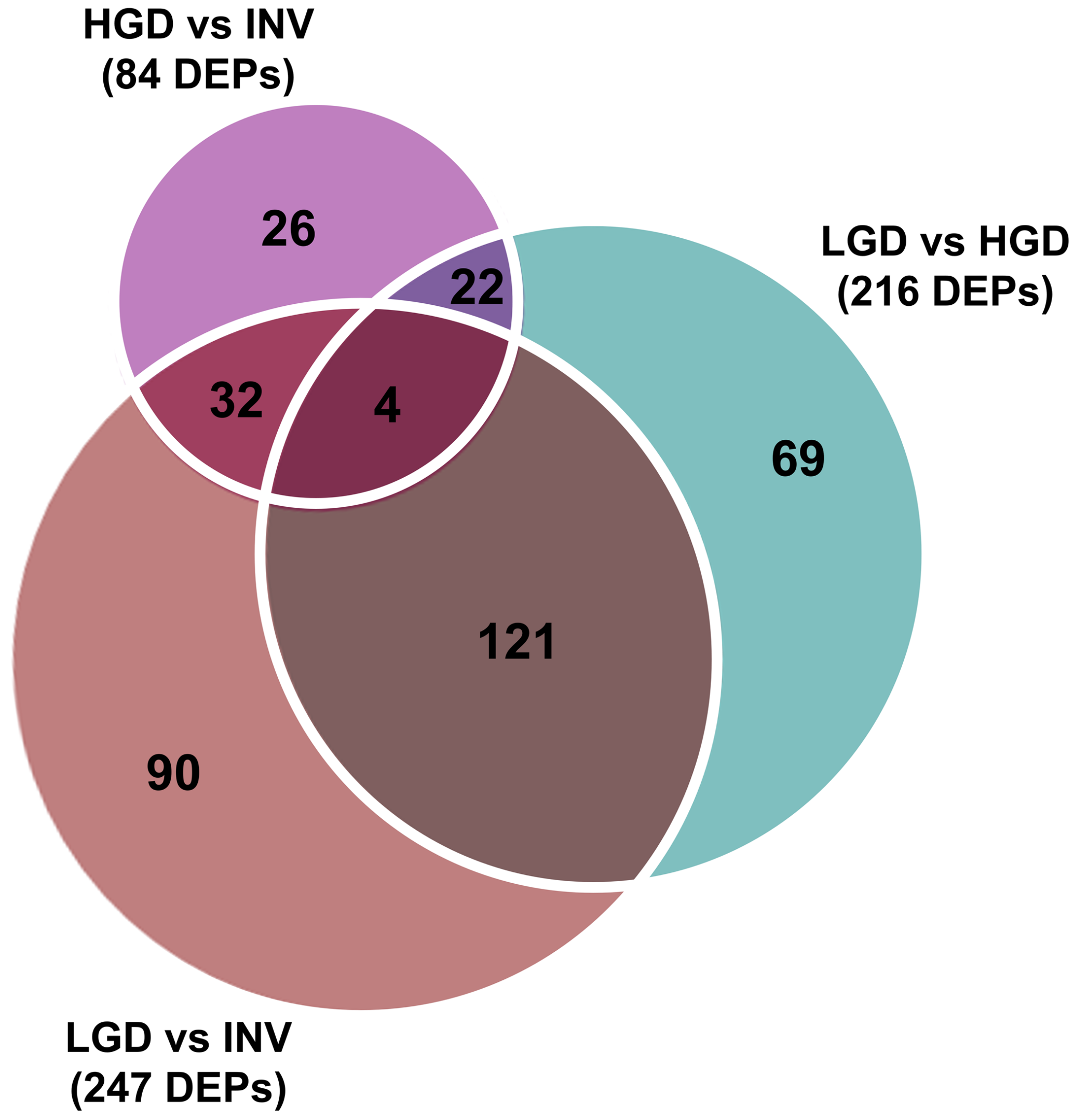


**Figure S8.** Venn diagram of differentially expressed proteins in 3 comparative groups. The statistical analysis (student’s t-test, *p* < 0.05) used in comparisons 1 (LGD versus HGD), 2 (HGD versus invasive IPMN), and 3 (LGD versus invasive IPMN) indicated that 216, 84, and 247 proteins were differentially expressed, respectively. Of the 364 DEPs, 179 were present in 2 or more comparative groups and were designated as the initial marker candidates. LGD, low-grade dysplasia; HGD, high-grade dysplasia; INV, invasive IPMN.


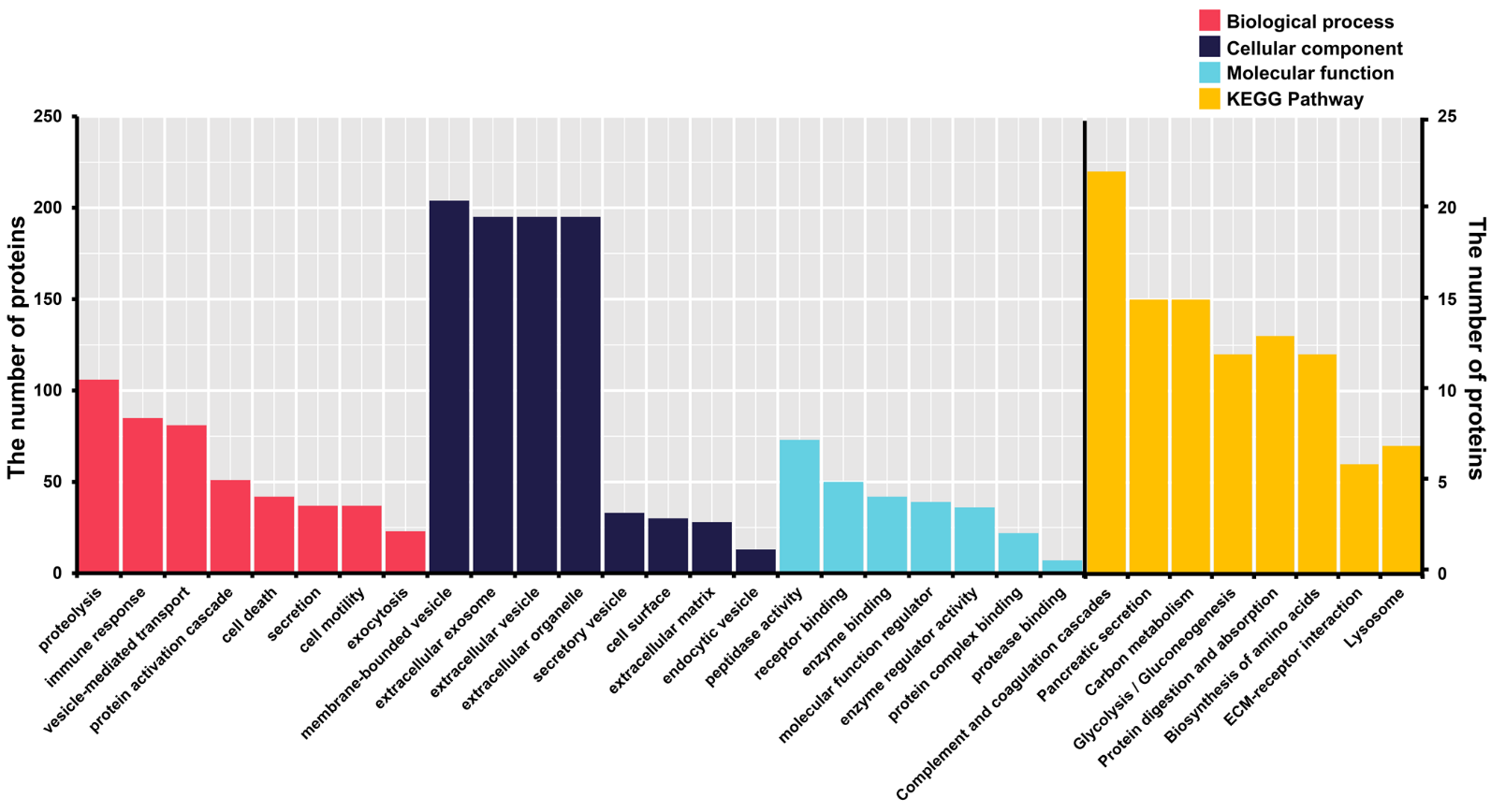


**Figure S9.** Results of Gene Ontology (GO) and KEGG pathway analyses. GO and KEGG pathway analyses were conducted using the DAVID bioinformatics tool. A total of 364 DEPs that originated from the statistical analysis between IPMN dysplasia were subjected to GO and KEGG pathway analyses. Each colored bar graph indicates the enriched terms in biological process (BP), cellular component (CC), molecular function (MF), and KEGG pathway. The number of participating proteins is shown on the left y-axis for GO terms and the right y-axis for KEGG pathway terms.


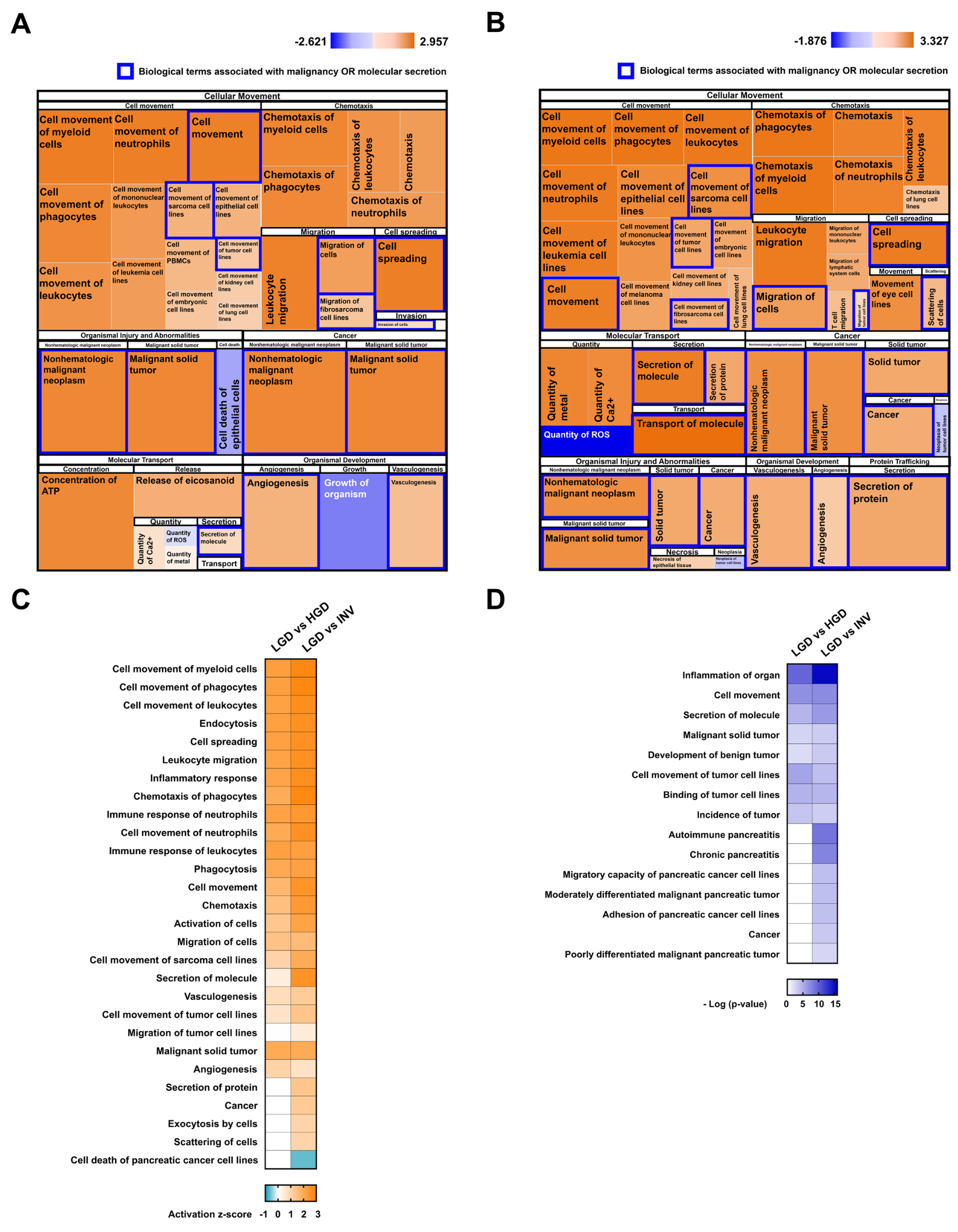


**Figure S10.** Ingenuity Pathway Analysis. Core analysis of IPA was conducted to evaluate the biological functions associated with the 216 DEPs of comparison 1 (LGD versus HGD) (A) and the 247 DEPs in comparison 3 (LGD versus invasive IPMN) (B). The heat maps indicate the upregulated and downregulated biological functions. The z-score of each biological function is represented by the sizes and colors of the boxes. Larger boxes signify higher z-scores. Orange indicates positive z-scores, and blue denotes negative z-scores. (C, D) Comparative analysis of IPA, visualizing the diseases and biological function terms across multiple analyses (comparisons 1 and 3) simultaneously. (C) The disease and biological function terms related to malignancy and molecular secretion were highly expressed in both comparisons (– 0.653 < z-score < 2.797). (D) Pancreas-specific diseases and cancer were significantly associated with the DEPs of comparison 3 (0 < - log10 of Fisher exact test p-value < 15.489). LGD, low-grade dysplasia; HGD, high-grade dysplasia; INV, invasive IPMN.


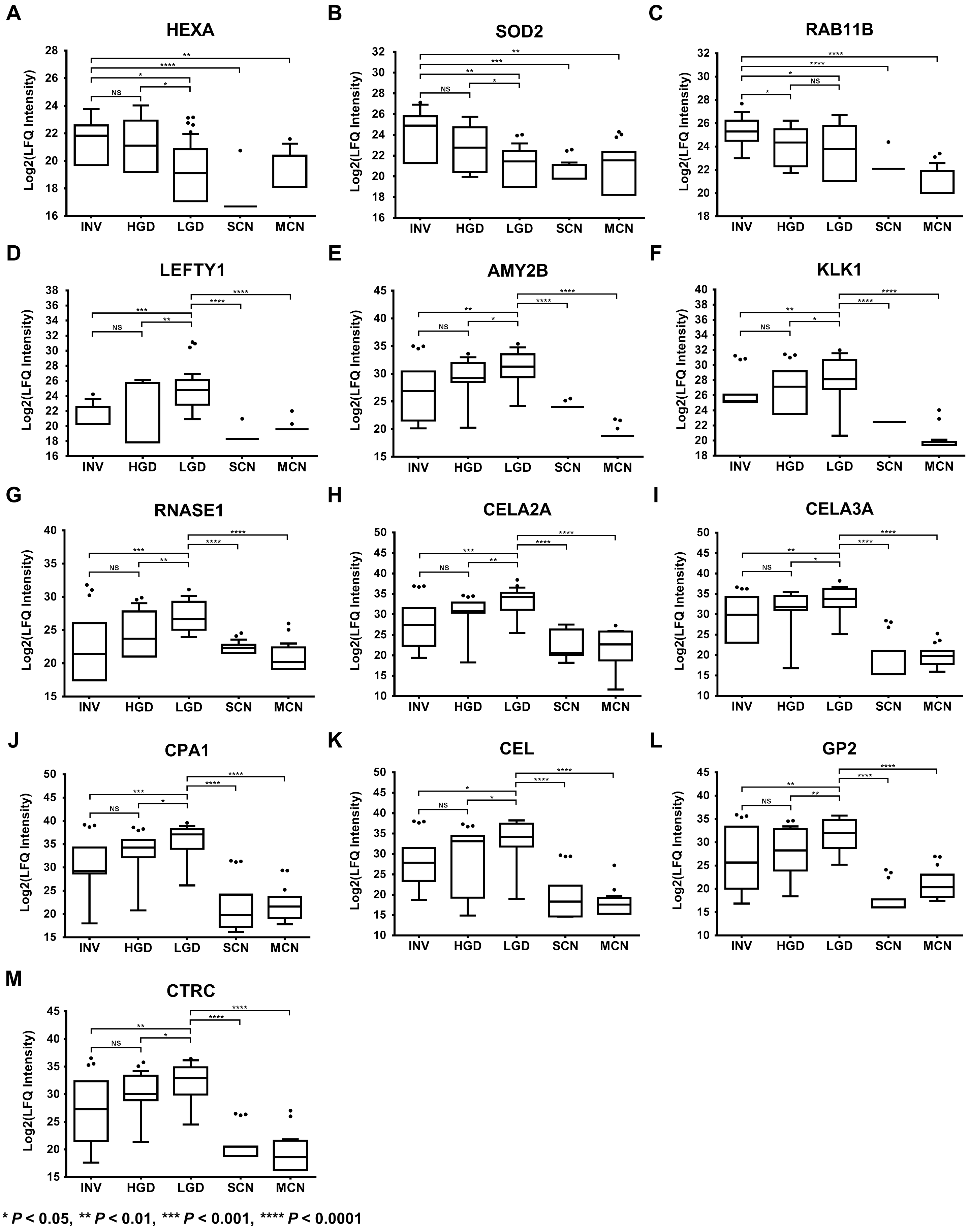


**Figure S11.** Thirteen potential biomarkers with expression patterns that were consistent with the degree of IPMN malignancy. The proteins indicated in box plot (A–C) are predominantly expressed in invasive IPMN. These upregulated potential markers were statistically significant in comparisons 6 (SCN versus invasive IPMN) and 7 (MCN versus invasive IPMN). The proteins indicated in box plot (D–M) are predominantly expressed in LGD. These downregulated potential markers were statistically significant in comparisons 4 (SCN versus LGD) and 5 (MCN versus LGD). LGD, low-grade dysplasia; HGD, high-grade dysplasia; INV, invasive IPMN; MCN, mucinous cystic neoplasm; SCN, serous cystic neoplasm; *, *p* < 0.05; **, *p* < 0.01; ***, *p* < 0.001; ****, *p* < 0.0001; NS, not available.


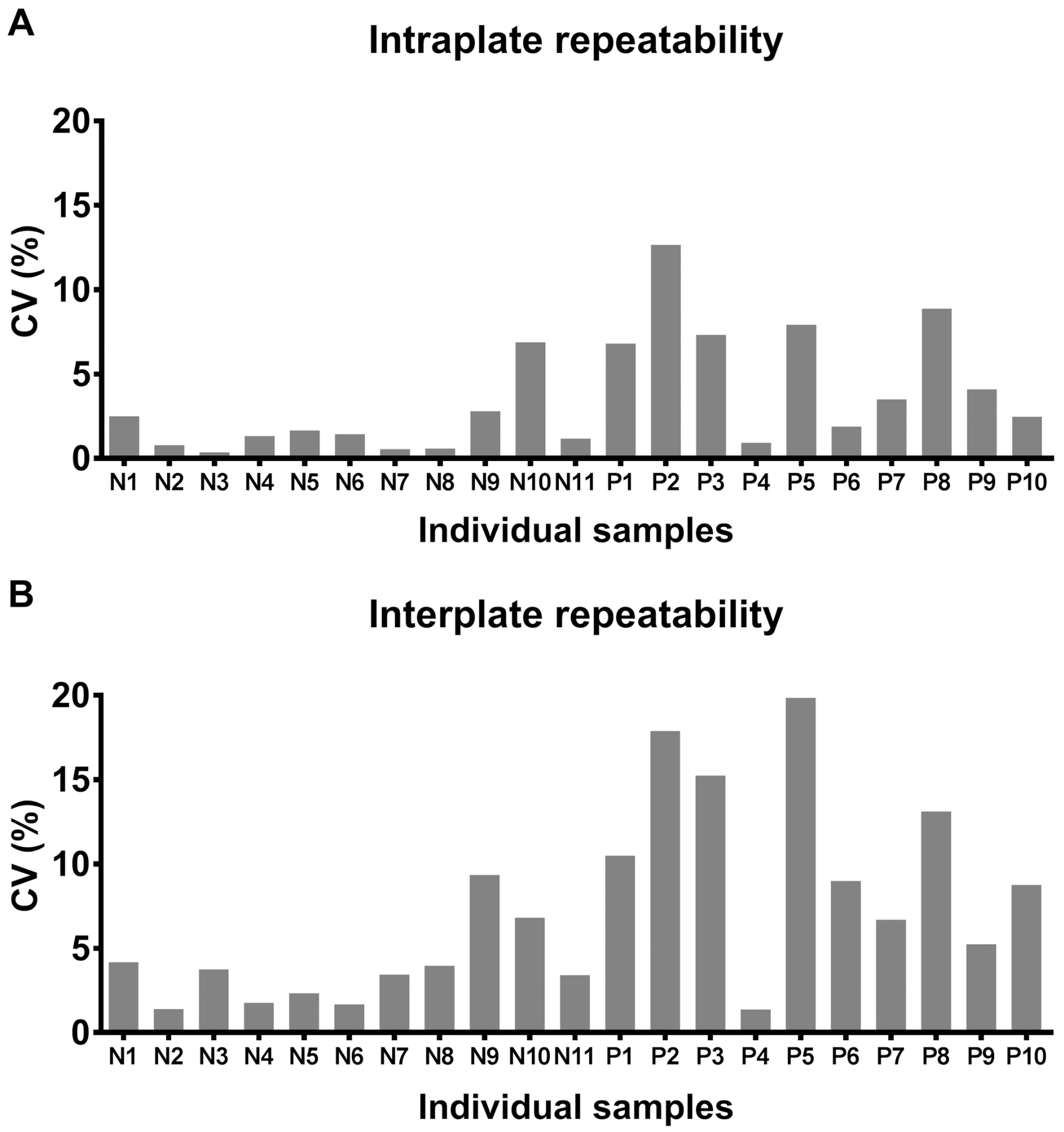


**Figure S12.** Intraplate and interplate repeatability of CD55 by ELISA. The coefficient of variation values of 3 replicates of negative controls (N1–N11) and positive controls (P1–P10) in intraplate and interplate repeatability. (A) Intraplate repeatability was measured by using 3 replicates for each of the 21 control samples on a single plate. (B) Interplate repeatability was evaluated by operating 3 plates, including 21 control samples at different times.


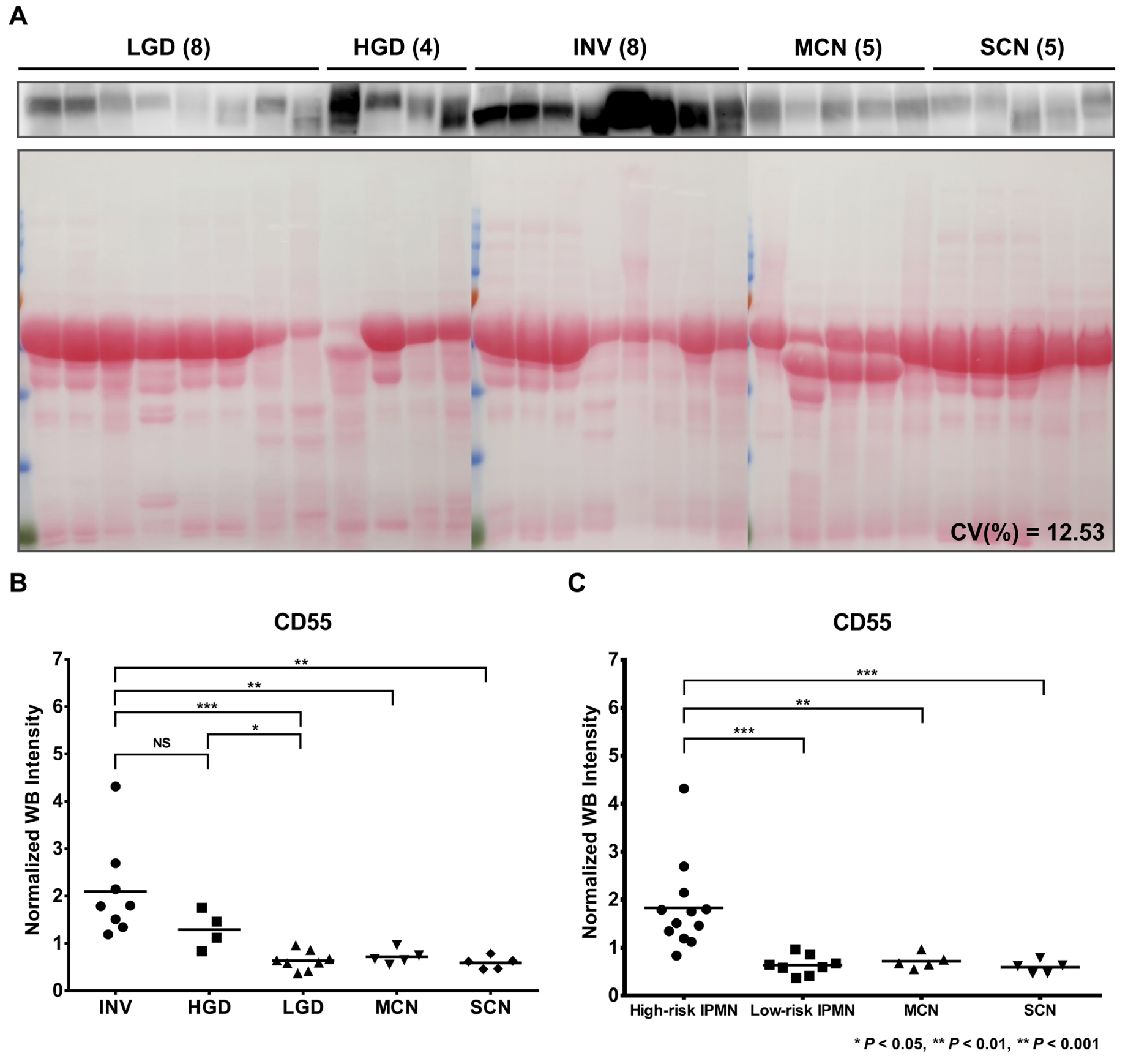


**Figure S13.** Validation of CD55 as a potential marker by Western blot. (A) Western blot band of CD55, with Ponceau S staining used as a loading control. The scatter dot plots of signal intensities are indicated according to two types of IPMN classification (B and C). LGD, low-grade dysplasia; HGD, high-grade dysplasia; INV, invasive IPMN; MCN, mucinous cystic neoplasm; SCN, serous cystic neoplasm; *, *p* < 0.05; **, *p* < 0.01; ***, *p* < 0.001; NS, not available.


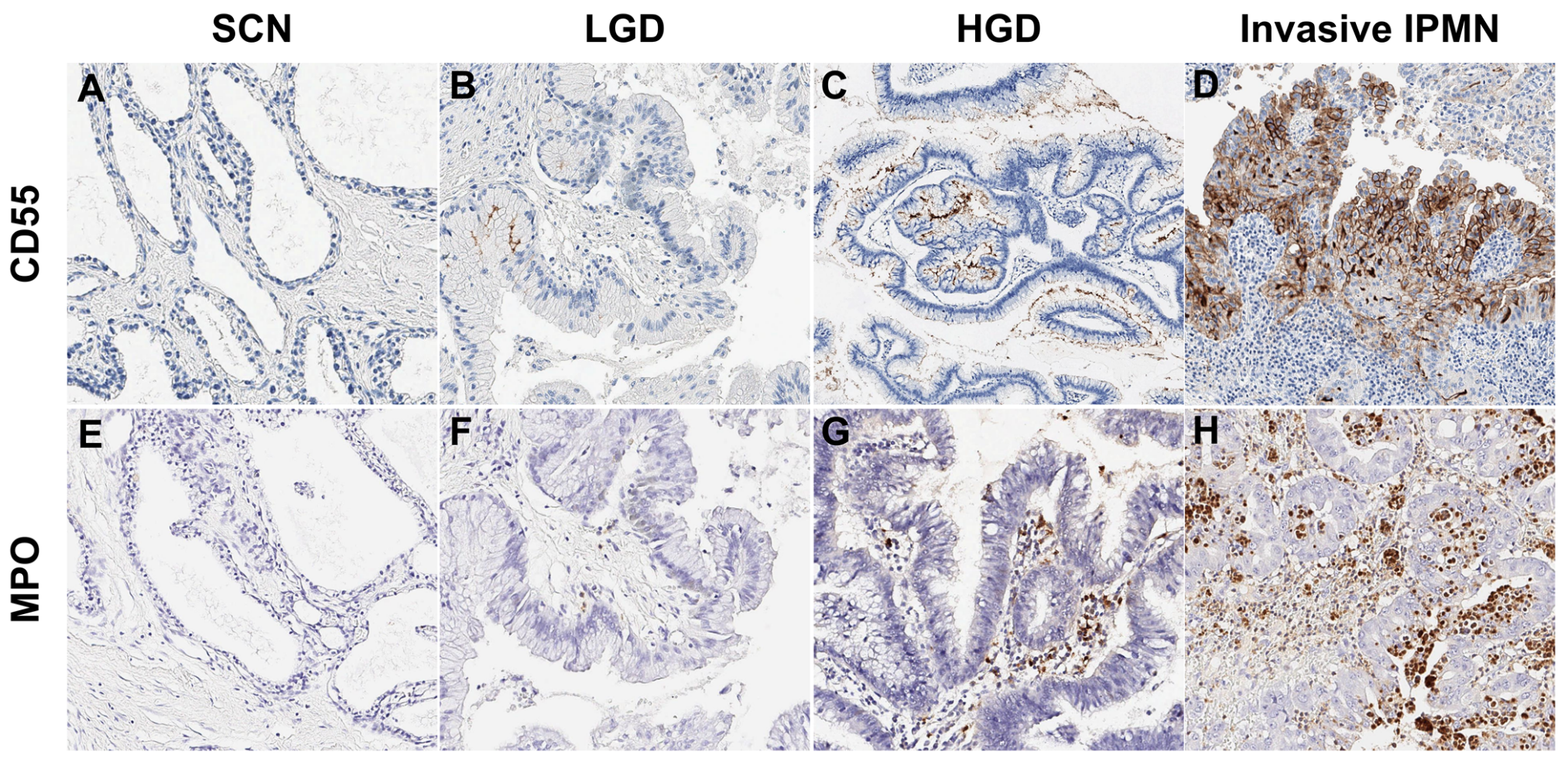


**Figure S14.** Immunohistochemical staining of CD55 and MPO. CD55 expression in serous cystic neoplasm (A), low-grade dysplasia (B), high-grade dysplasia (C), and invasive IPMN (D). Neutrophil infiltration in serous cystic neoplasm (E), low-grade dysplasia (F), high-grade dysplasia (G), and invasive IPMN (H). A–D: CD55 immunohistochemistry, E–H: myeloperoxidase (MPO) immunohistochemistry, original magnification x400. SCN, serous cystic neoplasm; LGD, low-grade dysplasia; HGD, high-grade dysplasia.

References

1. Do, M.; Han, D.; Wang, J.I.; Kim, H.; Kwon, W.; Han, Y.; Jang, J.Y.; Kim, Y. Quantitative proteomic analysis of pancreatic cyst fluid proteins associated with malignancy in intraductal papillary mucinous neoplasms. *Clin. Proteomics* **2018**, *15*, 17, doi:10.1186/s12014-018-9193-1.

2. Han, D.; Jin, J.; Woo, J.; Min, H.; Kim, Y. Proteomic analysis of mouse astrocytes and their secretome by a combination of FASP and StageTip-based, high pH, reversed-phase fractionation. *Proteomics* **2014**, *14*, 1604–1609, doi:10.1002/pmic.201300495.

3. Wisniewski, J.R.; Gaugaz, F.Z. Fast and sensitive total protein and Peptide assays for proteomic analysis. *Anal. Chem.* **2015**, *87*, 4110–4116, doi:10.1021/ac504689z.

4. Rappsilber, J.; Mann, M.; Ishihama, Y. Protocol for micro-purification, enrichment, pre-fractionation and storage of peptides for proteomics using StageTips. *Nat. Protoc.* **2007**, *2*, 1896–1906, doi:10.1038/nprot.2007.261.

5. Han, D.; Moon, S.; Kim, Y.; Kim, J.; Jin, J.; Kim, Y. In-depth proteomic analysis of mouse microglia using a combination of FASP and StageTip-based, high pH, reversed-phase fractionation. *Proteomics* **2013**, *13*, 2984–2988, doi:10.1002/pmic.201300091.

6. Park, J.; Han, D.; Do, M.; Woo, J.; Wang, J.I.; Han, Y.; Kwon, W.; Kim, S.W.; Jang, J.Y.; Kim, Y. Proteome characterization of human pancreatic cyst fluid from intraductal papillary mucinous neoplasm by liquid chromatography/tandem mass spectrometry. *Rapid Commun. Mass Spectrom.* **2017**, *31*, 1761–1772, doi:10.1002/rcm.7959.

7. Cox, J.; Mann, M. MaxQuant enables high peptide identification rates, individualized p.p.b.-range mass accuracies and proteome-wide protein quantification. *Nat. Biotechnol.* **2008**, *26*, 1367–1372, doi:10.1038/nbt.1511.

8. Cox, J.; Neuhauser, N.; Michalski, A.; Scheltema, R.A.; Olsen, J.V.; Mann, M. Andromeda: A peptide search engine integrated into the MaxQuant environment. *J. Proteome Res.* **2011**, *10*, 1794–1805, doi:10.1021/pr101065j.

9. Elias, J.E.; Gygi, S.P. Target-decoy search strategy for increased confidence in large-scale protein identifications by mass spectrometry. *Nat. Methods* **2007**, *4*, 207–214, doi:10.1038/nmeth1019.

10. Nesvizhskii, A.I.; Keller, A.; Kolker, E.; Aebersold, R. A statistical model for identifying proteins by tandem mass spectrometry. *Anal. Chem.* **2003**, *75*, 4646–4658.

11. Zhang, B.; Chambers, M.C.; Tabb, D.L. Proteomic parsimony through bipartite graph analysis improves accuracy and transparency. *J. Proteome Res.* **2007**, *6*, 3549–3557, doi:10.1021/pr070230d.

12. Tyanova, S.; Temu, T.; Cox, J. The MaxQuant computational platform for mass spectrometry-based shotgun proteomics. *Nat. Protoc.* **2016**, *11*, 2301–2319, doi:10.1038/nprot.2016.136.

13. Cox, J.; Hein, M.Y.; Luber, C.A.; Paron, I.; Nagaraj, N.; Mann, M. Accurate proteome-wide label-free quantification by delayed normalization and maximal peptide ratio extraction, termed MaxLFQ. *Mol. Cell Proteomics* **2014**, *13*, 2513–2526, doi:10.1074/mcp.M113.031591.

14. Kim, H.; An, S.; Lee, K.; Ahn, S.; Park, D.Y.; Kim, J.H.; Kang, D.W.; Kim, M.J.; Chang, M.S.; Jung, E.S.; et al. Pancreatic High-Grade Neuroendocrine Neoplasms in the Korean Population: A Multicenter Study. *Cancer Res. Treat.* **2020**, *52*, 263–276, doi:10.4143/crt.2019.192.

15. Hoffman, R.L.; Gates, J.L.; Kochman, M.L.; Ginsberg, G.G.; Ahmad, N.A.; Chandrasekhara, V.; Furth, E.E.; Vollmer, C.M.; Drebin, J.A. Analysis of cyst size and tumor markers in the management of pancreatic cysts: Support for the original Sendai criteria. *J. Am. Coll. Surg.* **2015**, *220*, 1087–1095, doi:10.1016/j.jamcollsurg.2015.02.013.

16. Chebib, I.; Yaeger, K.; Mino-Kenudson, M.; Pitman, M.B. The role of cytopathology and cyst fluid analysis in the preoperative diagnosis and management of pancreatic cysts >3 cm. *Cancer Cytopathol.* **2014**, *122*, 804–809, doi:10.1002/cncy.21460.

17. Bendtsen, J.D.; Jensen, L.J.; Blom, N.; Von Heijne, G.; Brunak, S. Feature-based prediction of non-classical and leaderless protein secretion. *Protein Eng. Des. Sel.* **2004**, *17*, 349–356, doi:10.1093/protein/gzh037.

18. Krogh, A.; Larsson, B.; von Heijne, G.; Sonnhammer, E.L. Predicting transmembrane protein topology with a hidden Markov model: Application to complete genomes. *J. Mol. Biol.* **2001**, *305*, 567–580, doi:10.1006/jmbi.2000.4315.

19. Petersen, T.N.; Brunak, S.; von Heijne, G.; Nielsen, H. SignalP 4.0: Discriminating signal peptides from transmembrane regions. *Nat. Methods* **2011**, *8*, 785–786, doi:10.1038/nmeth.1701.

20. Wilhelm, M.; Schlegl, J.; Hahne, H.; Gholami, A.M.; Lieberenz, M.; Savitski, M.M.; Ziegler, E.; Butzmann, L.; Gessulat, S.; Marx, H.; et al. Mass-spectrometry-based draft of the human proteome. *Nature* **2014**, *509*, 582–587, doi:10.1038/nature13319.

21. Nanjappa, V.; Thomas, J.K.; Marimuthu, A.; Muthusamy, B.; Radhakrishnan, A.; Sharma, R.; Ahmad Khan, A.; Balakrishnan, L.; Sahasrabuddhe, N.A.; Kumar, S.; et al. Plasma Proteome Database as a resource for proteomics research: 2014 update. *Nucleic Acids Res.* **2014**, *42*, D959–D965, doi:10.1093/nar/gkt1251.

22. Muthusamy, B.; Hanumanthu, G.; Suresh, S.; Rekha, B.; Srinivas, D.; Karthick, L.; Vrushabendra, B.M.; Sharma, S.; Mishra, G.; Chatterjee, P.; et al. Plasma Proteome Database as a resource for proteomics research. *Proteomics* **2005**, *5*, 3531–3536, doi:10.1002/pmic.200401335.

23. Schirle, M.; Heurtier, M.A.; Kuster, B. Profiling core proteomes of human cell lines by one-dimensional PAGE and liquid chromatography-tandem mass spectrometry. *Mol. Cell. Proteomics* **2003**, *2*, 1297–1305, doi:10.1074/mcp.M300087-MCP200.

24. Snozek, C.L.; Mascarenhas, R.C.; O'Kane, D.J. Use of cyst fluid CEA, CA19-9, and amylase for evaluation of pancreatic lesions. *Clin. Biochem.* **2009**, *42*, 1585–1588, doi:10.1016/j.clinbiochem.2009.06.020.

| 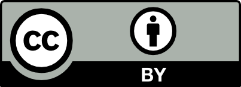 | © 2020 by the authors. Licensee MDPI, Basel, Switzerland. This article is an open access article distributed under the terms and conditions of the Creative Commons Attribution (CC BY) license (http://creativecommons.org/licenses/by/4.0/). |
| --- | --- |
